# Hospitalization Trends Due to Chronic Liver Diseases: Vicious Circle of Co-Morbidities and Hospitalization Length

**DOI:** 10.3390/clinpract16030057

**Published:** 2026-03-06

**Authors:** Ivana Pantic, Nikola Grubor, Sofija Lugonja, Nina Rajovic, Svetlana Miltenovic, Marija Brankovic, Tijana Gmizic, Tamara Milovanovic

**Affiliations:** 1 Clinic of Gastroenterology and Hepatology, University Clinical Center of Serbia, St. Dr Koste Todorovica 2, 11000 Belgrade, Serbia; ilic.ivana04@gmail.com; 2 Institute for Medical Statistics and Informatics, Faculty of Medicine, University of Belgrade, St. Dr Subotica 15, 11000 Belgrade, Serbia; nickgrubor@gmail.com (N.G.); nina94rajovic@gmail.com (N.R.); 3 Division of Gastroenterology, Department of Internal Medicine, General Hospital “Djordje Jovanovic”, St. Dr Vase Savica 5, 23000 Zrenjanin, Serbia; prolesofija@gmail.com; 4 Institute of Public Health of Belgrade, St. Bulevar despota Stefana 54, 11000 Belgrade, Serbia; svetlana.miltenovic@zdravlje.org.rs; 5 University Hospital Center Bezanijska Kosa, St. Bezanijska Kosa, 11000 Belgrade, Serbia; marijasbrankovic@gmail.com (M.B.);; 6 Faculty of Medicine, University of Belgrade, St. Dr Subotica 8, 11000 Belgrade, Serbia

**Keywords:** chronic liver disease, hospitalization, co-morbidity, cirrhosis

## Abstract

**Background and Aims**: Chronic liver diseases (CLD) represent a significant healthcare burden, mostly due to late diagnosis and numerous co-morbidities. We evaluated the effect of co-morbidities, cirrhosis, and disease etiology on hospitalization duration. **Methods**: Hospitalizations due to alcohol-related, viral, autoimmune, and overlapping liver disease in Belgrade, Serbia (2016–2022), were identified using pre-defined discharge codes. We investigated the hospitalization trend descriptively by plotting the relative mean change in the hospitalization length against time. Assuming the covariate relationship in the directed acyclic graph, we estimated the direct causal effect of the diagnosis type on the length of stay (LOS) by fitting pre-specified Bayesian distributional lognormal models based on domain knowledge. We conducted a post hoc analysis of the impact of cirrhosis on LOS per primary diagnosis. **Results**: The empirical data show a decrease in the estimated average LOS (8.25–5.51 days). For the same period, the median LOS decreased (4 days (IQR 0–12) to 1 day (IQR 1–7)). In 2021, the share of short-term hospitalizations rose to 46.94%, while the median long-term hospitalization peaked at 11.5 days (IQR 7–21). The expected LOS was the highest for the primary diagnosis of autoimmune liver disease (15.89, 95% CI [14.74, 17.2] days), followed by alcohol-related liver disease (14.22, 95% CI [13.68, 14.79] days). The largest impact of cirrhosis on LOS was observed among patients hospitalized due to viral disease (4.19, 95% CI [2.29, 6.33] days). **Conclusions**: The presence of co-morbidities and cirrhosis significantly affects LOS. In order to provide better treatment and reduce healthcare costs, there is the need to detect liver disease at earlier stages and better manage its associated co-morbidities.

## 1. Introduction

Chronic liver diseases (CLD) represent a significant global health burden. According to World Health Organization (WHO) data, end-stage liver disease, or cirrhosis, is considered the ninth-leading cause of death in Europe, which demonstrates its strong impact on the healthcare system [1]. The prevalence of CLD etiology varies according to the region. According to the latest data, alcohol-related liver disease remains the most prevalent CLD in Europe, as well as the most common etiology of liver cirrhosis [1,2], even though the WHO reported a decrease in the total per capita alcohol consumption in the world population [3]. However, despite the decrease in total alcohol consumption, the estimated prevalence of specific alcohol-use patterns in current drinkers, such as “binge drinking” (consuming at least 60 g of pure alcohol on one or more occasions in the last month), is still as high as 38% percent [3]. Finally, the evolution of harmful patterns of alcohol use led to the recent introduction of the term “high-intensity drinking”, which is defined as alcohol intake at levels twice or more the sex-specific threshold for binge drinking and is increasingly common in younger age groups [4]. While its association with imminent severe health and safety consequences is expected, its long-term effects and global prevalence are yet to be evaluated.

Additionally, a significant increase in metabolic dysfunction-associated steatotic liver disease (MASLD) prevalence has been consistently reported both in adults and children, threatening to become the leading cause of CLD globally [1,2,5]. Furthermore, the increasing rates of obesity and type 2 diabetes, both strong risk factors associated with this clinical entity, are highly concerning [1,2,5]. Given the rising prevalence of the aforementioned conditions, together with hazardous alcohol use [6,7,8], it is no surprise that the prevalence of both CLD and cirrhosis has increased during the previous years in the Western world [2]. Furthermore, it is not uncommon for patients treated in hepatology clinics to have multiple co-morbidities, which could be explained by population growth and aging, in addition to the systemic effects of different CLDs [9,10,11]. Given the increased CLD prevalence, a lack of widespread healthcare policies, and numerous co-morbid conditions, one could expect an increase in mortality, hospitalizations, and healthcare-related costs in years to come.

Because of its significant burden, several studies were conducted in order to quantify CLD-related mortality and hospitalization trends, especially in the context of the recent Coronavirus disease (COVID-19) pandemic [12,13,14]. In general, recent data implicate a reduction in the length of stay and in-hospital mortality during the previous years in patients diagnosed with cirrhosis [14,15,16,17], which could be attributed to better treatment strategies, earlier disease detection and the widespread application of official clinical practice guidelines.

However, despite the aforementioned, the exact data, or at least estimates regarding cirrhosis morbidity and mortality, are usually lacking at national levels. Therefore, this population-based study examined the temporal trends of hospitalizations due to CLD in Belgrade, Serbia, from 1 January 2016 to 31 December 2022. We also aimed to evaluate the effect of several co-morbidities, the presence of cirrhosis, and disease etiology on hospitalization duration.

## 2. Methods

### 2.1. Healthcare System Organization

The Republic of Serbia healthcare system operates on compulsory health insurance and is based on the Bismarck model. Healthcare funding is primarily derived from mandatory payroll contributions and is administered centrally by the Republic Health Insurance Fund, implying that the state practices a strong governance role in its organization and functioning [18]. In addition, the Serbian healthcare system does not impose restrictive insurance-based eligibility barriers on access to essential healthcare services for insured individuals. However, patients still report notably high out-of-pocket payments [18,19]. Healthcare delivery in Serbia is structured across three highly structured hierarchical levels, primary, secondary, and tertiary care, with primary care being delivered through community health centers, serving as the first point of contact, while secondary and tertiary healthcare centers offer in- and outpatient treatment in general hospitals and highly specialized clinical centers, respectively [19]. In addition to the large public facilities network, recent years have witnessed a significant and growing private healthcare sector, which is mainly directed at providing outpatient services rather than inpatient treatment [19].

### 2.2. Study Design

#### 2.2.1. Study Population

We obtained serial data for 2016–2022 (1 January 2016 to 31 December 2022) from the Institute of Public Health of Belgrade, which contain data regarding all public health services provided for the territory of Belgrade, Serbia’s capital. The dataset included length of hospitalization, age, gender, diagnostic codes, primary diagnosis of referral, and discharge type. A researcher independent of the analyst reviewed the diagnostic codes for consistency. The complete study population and descriptive statistics stratified by primary diagnosis are shown in Table 1.

#### 2.2.2. Inclusion Criteria

We evaluated individual hospitalization episodes of adult patients (age > 18 years) due to alcohol-related, viral, autoimmune, and overlapping liver disease. Hospitalization episodes were identified using primary and secondary discharge diagnosis codes based on the 10th revision of the International Classification of Diseases (ICD-10). In our dataset, the primary diagnosis specifies the acute principal reason for admission. Secondary diagnosis codes explicitly capture chronic, pre-existing co-morbidities present on admission, rather than acute hospital-acquired complications. This structure allows us to evaluate baseline disease burden while mitigating the risk of reverse causation from nosocomial events. During the data curation process, CLD-related discharge diagnosis codes were classified into two lists: (A) alcohol-associated hepatitis (K70.1), liver fibrosis or cirrhosis due to alcohol-related liver disease (K70.2, K70.3), liver failure due to alcohol-related liver disease (K70.4), alcohol-related liver disease in general (K70.9), hepatitis B and hepatitis C virus infection (B18.1, B18.2), primary biliary cholangitis (K74.3), and autoimmune hepatitis (K75.4); (B) acute and chronic liver failure (K72.0, K72.1) and unspecified liver cirrhosis (K74.6). Those with a discharge code from List A were automatically selected and further classified into viral, autoimmune, alcohol-related, or overlapping liver disease. The term “overlapping” liver disease was used in those cases in which more than one liver disease was coded (e.g., patients with alcohol-related and viral liver disease). Afterward, those with a discharge code from List B and at least one discharge code from List A were also selected and classified into the groups mentioned above based on the stated liver disease etiology. Hospitalization reports which contained SARS-CoV2 infection-related admission codes were not included in the analyses.

#### 2.2.3. Ethical Approval

This study is based exclusively on the secondary analysis of publicly available, aggregated health statistical data collected by the Statistical Office of the Republic of Serbia. Data collection adhered to strict ethical guidelines ensuring the confidentiality and privacy of all participants. The data used does not contain personal or identifiable information, and individual subjects cannot be identified. Given that the research involves secondary analysis of anonymized, publicly available data, approval from the institutional ethics committee and informed consent are not required, which is in compliance with the Law on Official Statistics of the Republic of Serbia.

#### 2.2.4. Hospitalization Trends and Impact of Co-Diagnosis

We investigated the hospitalization trend descriptively by plotting the relative mean change in the hospitalization length against time. We also compute the median length of stay alongside its interquartile range (IQR) as a more robust measure of the typical hospitalization length. Based on domain knowledge, we hypothesized that two data-generating processes form an overall length-of-stay trend. The first relates to short-term (<24 h) hospitalizations, which are often of a diagnostic form and should represent a more stable cohort of patients. The second is the long-term hospitalization cohort, representing patients with more severe disease who have been admitted to the hospital ward for more intensive treatment or thorough diagnostic work-up. We decomposed the primary trend into the former to investigate any systematic changes.

**Figure 1:**
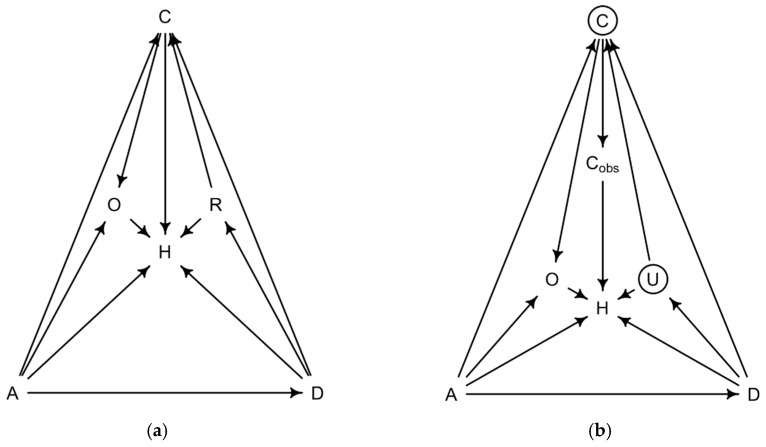
Since the actual rate of co-morbidities is unobserved, and only the number of co-diagnoses is known, the physician is a possible confounder. Suppose the “effect” of the physician determines a significant portion of the length of stay. This will bias the estimate depending on her vigilance in detecting and documenting co-morbidities and risk aversion. A, age; D, primary diagnosis; C, co-diagnosis; R, cirrhosis; Cobs, observed co-diagnosis; H, hospitalization length; O, outcome; U, unobserved confound. (**a**) The analysis assumes the following directed acyclic graph. (**b**) Possible unobserved confounders.

Assuming the covariate relationship in the directed acyclic graph (DAG), we estimated the direct causal effect of diagnosis type on the length of hospital stay (Figure 1) [20]. To utilize the maximum information available, we pre-specified several Bayesian distributional lognormal models based on domain knowledge. The effect of age is known to be non-linear; to avoid overfitting, we opted to model age with a restricted cubic spline with a pre-specified number of knots (five). An interaction effect was also considered between the primary diagnosis and the number of co-diagnoses, since different underlying conditions tolerate differing co-diagnoses. Additionally, we tested the estimated predictive power of assuming that, as the number of co-diagnoses grows, so does the length of hospitalization, compared with allowing the co-diagnosis to take a non-linear form within each primary diagnosis. Therefore, we assessed a complete linear additive model, a model with age modeled with a non-linear restricted cubic spline, co-diagnoses modeled as a monotonically increasing relationship with the outcome, and finally an interaction model between monotonically modeled co-morbidity number and type of diagnosis. Model comparison was done via Pareto-smoothed importance sampling leave-one-out cross-validation (PSIS). We present the posterior distributions of the average hospitalization length by primary diagnosis to communicate the complete uncertainty of the estimate. The posterior distribution of the relationship between co-diagnosis and length of hospitalization, our main target of inference, is shown separately.

Additionally, we conducted a post hoc analysis of the impact of cirrhosis on the length of stay per primary diagnosis. We define cirrhosis as the presence of ICD-10 codes for cirrhosis of any cause. To properly account for the difference in length of hospitalization and communicate the uncertainty completely, we plot posterior contrasts between cirrhotic and non-cirrhotic patients.

### 2.3. Statistical Analysis

We estimated the posterior distribution using Hamiltonian Markov Chain Monte Carlo (MCMC), as implemented in Stan version 2.32.2 [21]. When deriving point estimates from the posterior distributions, we use the median. We assessed convergence through the inspection of trace plots and R-hat values, which should be below 1.01 [22], and effective sample size (ESS), which should be greater than 1000 [23]. Model out-of-sample predictive performance was assessed using leave-one-out cross-validation approximated by Pareto-smoothed importance sampling [24]. Priors were chosen to be weakly informative through prior predictive simulation so that pre-data predictions span the range of scientifically plausible outcomes. Additionally, posterior predictive checks and leave-one-out probability integral transform (LOO-PIT) calibration plots were used to assess model fit [25]. The MCMC trace plot is visualized in Figure S1. The LOO-PIT plot is shown in Figure S2.

The statistical analysis was done in R version 4.3.2 [26]. The packages utilized were: brms, marginaleffects, ggdist, rethinking and dagitty [23,27,28,29,30].

## 3. Results

### 3.1. Study Population

The analysis included 3975 hospitalizations. The study population consisted of a significant number of short-term hospitalizations, defined as lasting a maximum of 24 h (41% of all data). These short-term hospitalizations belong to a separate data-generating process compared to patients who have been hospitalized for longer. Therefore, we limited our mediation analysis to the long-term hospitalization population.

**Figure 2:**
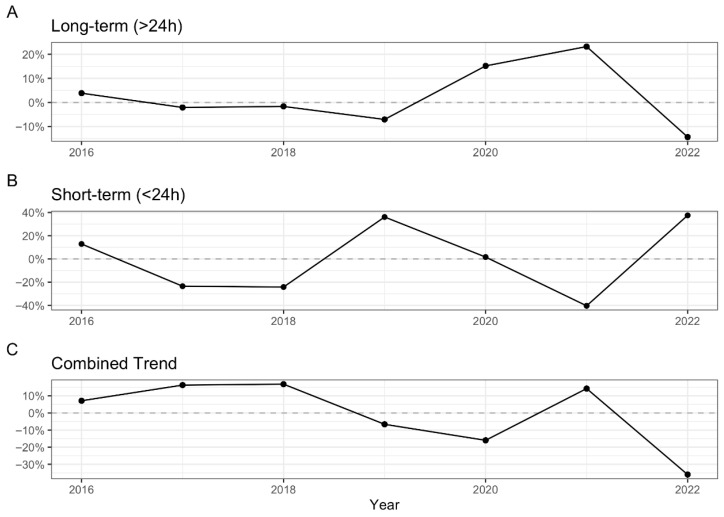
Relative mean-change in average hospitalization length (2016–2022). The overall combined trend (**C**) is decomposed to illustrate the divergent underlying patterns of (**A**) long-term (>24 h) and (**B**) short-term (<24 h) hospitalizations. The y-axis represents the percentage change relative to the grand mean for each respective category. Notably, the combined trend masks significant behavioral shifts during the 2020–2021 period, where long-term hospitalizations experienced a steady increase, inversely mirroring a sharp decline in short-term, daily hospitalizations before both trends reversed in 2022.

The empirical data show an overall decrease in the length of stay during 2016–2022 from an estimated average of 8.25 to 5.51 days. For the same period, the median hospitalization length decreased from 4 days (IQR 0–12) to 1 day (IQR 1–7). This trend can be decomposed into two parts: the short-term and long-term hospitalizations. The relative share of short-term hospitalizations and day hospital visits decreased from 38.15% to 26.33% from 2016 to 2018, respectively. The median long-term hospitalization length remained relatively constant at 9 days (IQR 4–17) during the same period. In 2021, the share of short-term hospitalizations rose to 46.94%, while the median long-term hospitalization peaked at 11.5 days (IQR 7–21) before returning to baseline. Thus, when the time spent in the hospital was longest, the number of patients actually hospitalized was low. The corresponding inverse relationship with long-term hospitalizations can be seen in Figure 2, where we plot the year-by-year percent change in the observed trends.

The long-term hospitalized population is summarized in Table 1.

### 3.2. Impact of Diagnosis and Co-Diagnosis

**Figure 3:**
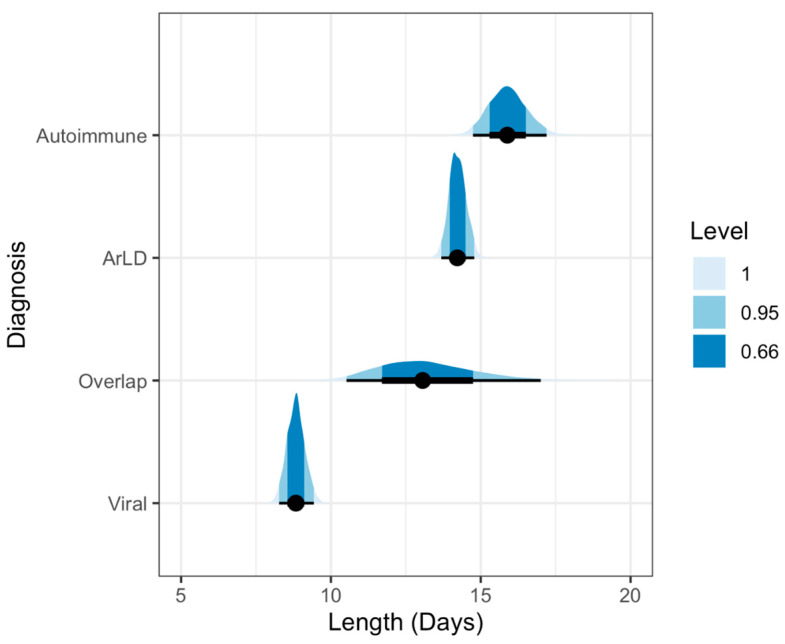
Posterior predictive distributions of the expected length of hospital stay stratified by primary liver disease diagnosis. The plot visualizes the estimated average hospitalization length (in days) for each etiology, derived from the primary Bayesian lognormal model. The black dots represent the median point estimates, while the thick and thin black lines represent the 66% and 95% credible intervals, respectively. The shaded blue density curves illustrate the full probability distribution of the estimates. Autoimmune liver disease and alcohol-related liver disease (ArLD) exhibit the longest expected hospitalizations with high model precision. In contrast, viral hepatitis is associated with a markedly shorter expected stay. The notably wider probability distribution for overlap syndrome reflects the higher degree of uncertainty and clinical heterogeneity within this specific patient subgroup.

**Figure 4:**
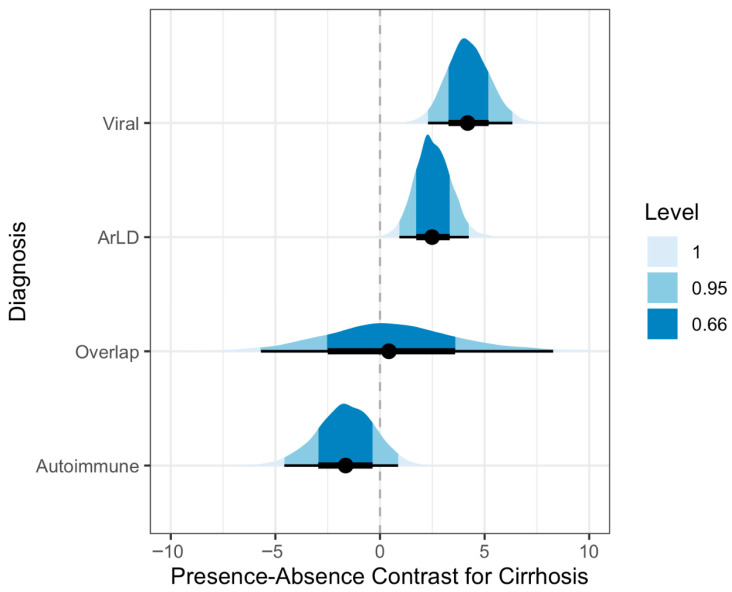
Posterior predictive distributions of the effect of cirrhosis on length of hospital stay, stratified by primary liver disease diagnosis. The plot displays the estimated difference in hospitalization length (in days) between patients with and without cirrhosis. The vertical dashed line at zero represents no difference. Black dots indicate the median estimated contrast, while the thick and thin horizontal black lines denote the 66% and 95% credible intervals, respectively. Shaded areas represent the full posterior probability density. Positive values indicate that the presence of cirrhosis is expected to prolong the hospital stay. Notably, concurrent cirrhosis significantly increases the expected length of stay for patients with viral hepatitis and alcohol-related liver disease (ArLD). Conversely, cirrhosis is associated with a slightly shorter expected stay for patients with autoimmune liver disease. The highly dispersed distribution for overlap syndrome crosses the zero-line, indicating substantial uncertainty regarding the clinical impact of cirrhosis on hospitalization length within this specific subgroup.

The expected hospital stay was the highest for the primary diagnosis of autoimmune liver disease (15.89, 95% CI [14.74, 17.2] days), followed by alcohol-related liver disease (14.22, 95% CI [13.68, 14.79] days). We show the average empirical length of hospitalization by the type of diagnosis in Table 1. The expected average hospitalization stratified by diagnosis is shown in Table 2. In order to communicate the total uncertainty in the estimates, we show the posterior distributions of the expected mean hospitalization per primary diagnosis in Figure 3. The expected posterior contrast between patients with and without cirrhosis is shown in Figure 4. The most considerable difference of 4.19 (95% CI [2.29, 6.33]) days was observed between patients whose primary diagnosis was viral hepatitis. The highest negative difference of −1.64 (95% CI [−4.57, 0.87]) days was observed among patients with the primary diagnosis of autoimmune liver disease. The lowest difference was observed among patients with the primary diagnosis of overlap syndrome (0.42, 95% CI [−5.69, 8.28]), albeit with a wide credible interval.

**Figure 5:**
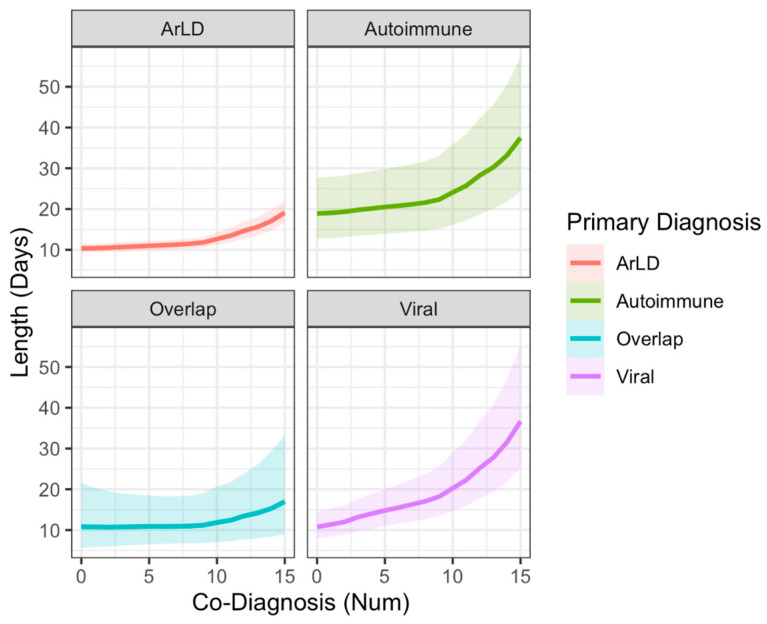
Estimated marginal effects of cumulative co-diagnosis burden on hospital length of stay, stratified by primary liver disease diagnosis. The plot visualizes the interaction between the number of chronic co-diagnoses and the primary etiology, derived from the Bayesian lognormal model. Solid lines represent the expected average length of hospitalization (in days) at varying levels of co-morbidity burden, while the shaded regions indicate the 95% credible intervals. Across all primary diagnoses, an accumulating number of co-diagnoses corresponds with a prolonged hospital stay; however, the magnitude and trajectory of this effect differ by etiology. For alcohol-related liver disease (ArLD) and overlap syndrome, the expected length of stay remains relatively stable at lower co-diagnosis counts before curving upward at higher thresholds. In contrast, hospitalizations driven by viral hepatitis exhibit a much steeper, more acute increase in length of stay as co-morbidities accumulate. Autoimmune liver disease maintains the highest expected baseline length of stay across all co-morbidity levels, though predictions become highly uncertain at the upper extremes due to data sparsity.

The relationship between disease burden and length of hospitalization is shown in Figure 5. At low numbers of co-diagnoses, the length of stay remained relatively stable for alcohol-related liver disease, autoimmune hepatitis, and overlap syndrome, more severely impacting the length of hospitalization at higher ends of the scale. For viral hepatitis, the impact of the co-diagnosis number seems more significant, increasing the slope of the effect more acutely.

The model with the best out-of-sample performance (based on PSIS score) was the one that included the interaction between monotonically modeled co-diagnoses and type of diagnosis, with age modeled as a simple linear relationship. Additionally, the model with monotonic assumptions for co-diagnosis had the highest estimated predictive power, with the highest effect occurring at a different cutoff depending on the primary diagnosis. The effect of co-diagnosis on increased length of stay when the primary diagnosis of viral hepatitis was established occurred in three co-diagnoses, while the other primary diagnoses peaked at ten.

## 4. Discussion

This study investigated hospitalization trends descriptively for several CLDs (ArLD, viral, autoimmune, and “overlapping”) from 2016 to 2022. We have also estimated the direct causal effect of the CLD etiology, number of co-diagnoses, and presence of cirrhosis on the length of hospital stay. To the best of the authors’ knowledge, this is the first study that addresses hospitalization trends of CLD in this manner, by explicitly modeling the generative structure of the data.

According to our results, the long-term hospitalization length remained relatively stable until 2019, only returning to baseline in 2022. There was a significant increase in short-term hospitalizations during 2019 but no increase in long-term hospitalization length. As the long-term hospitalization length increased over the same period, short-term hospitalizations fell below their average, possibly reflecting the impact of the pandemic. The pandemic could impact hospitalization trends in at least two different ways. Firstly, it is well known that the pandemic led to significant healthcare disruptions and the cessation of standard medical care because of the reasonable need for the prioritization of acutely ill patients [31,32,33]. This mechanism could explain a decrease in short-term hospitalizations, which in our country mainly represent outpatient visits during which invasive procedures are performed or specific treatment is administered (e.g., diagnostic upper endoscopy, therapeutic large-volume paracentesis, albumin administration). On the other hand, since patients delayed seeking medical advice due to social distancing measures and presented themselves in the advanced stages of their disease, the pandemic resulted in a more significant number of patients requiring intensive healthcare support, which in turn increased the length of long-term hospitalization [34]. In 2022, long-term hospitalization length fell, and we observe a concurrent rise in short-term hospitalization as the effects of the pandemic wane and previous healthcare practices return.

Additionally, we reported a global decreasing trend in the length of stay during the observed years from an estimated average of 8.25 to 5.51 days, concordant with the results of several previously published studies [14,15,17]. Moreover, after analyses of the expected average hospitalization duration per CLD etiology (Figure 3), our results showed that the highest hospitalization length was observed in autoimmune liver diseases, followed by ArLD. These results are only partially in concordance with those previously published since the mean hospital stay of patients admitted with ArLD cirrhosis was significantly longer than that of patients diagnosed with cirrhosis of other etiology [14]. Interestingly, our data also show significantly longer hospitalizations in general, irrespective of the CLD etiology. Our data suggest hospitalization durations > 10 days, while other authors mainly report a length of stay < 10 days, especially in the context of decreasing hospitalization length trends [14,34]. Both results could be explained by the lack of strict control of excessive medical costs and the subsequent lack of limiting measures in delivering medical services of any kind. This lack of checks and balances directly results from our national healthcare system organization, which relies mainly on public insurance measures and solidarity.

Regarding the effect of several co-diagnoses on the length of stay, we observed that, at low numbers of co-diagnoses, the length of stay remains relatively stable while more severely impacting the length of hospitalization at higher ends of the scale. The differential effect of the co-diagnosis number on the underlying cause of hospitalization could be explained by the higher mortality risk in specific disease subgroups. For example, patients who have already developed liver failure cannot tolerate higher numbers of co-occurring disease states. However, as the number of co-diagnoses accumulates, the length of hospital stays starts to increase dramatically and at a faster rate for infectious diseases than other types of hospitalization (Figure 5). Interestingly, the primary diagnosis of viral hepatitis seems more sensitive to the rising number of co-diagnoses. Given that viral CLD tends to remain asymptomatic for a long period, it is not uncommon for the diagnosis to be established after the occurrence of either complications or extra-hepatic manifestations of the disease. Therefore, in the setting of late diagnosis and unavailability of effective antiviral treatment (which is no longer the case in our country), it is not uncommon for these patients to rapidly deteriorate after complications arise, which reflect a more vulnerable disease state. In addition, other primary diagnoses exhibit a rise in hospitalization only later when a more significant number of co-diagnoses has accumulated. This delayed risk increase could be due to many common diagnostic labels that do not immediately impact hospitalization length (e.g., hypertension, anemia, etc.). Finally, another explanation could be that patients with more severe disease die before ever having the possibility to have more conditions documented.

Patients with cirrhosis are a particularly vulnerable population, suffering from debilitating complications. Therefore, the posterior contrasts relating to cirrhosis show that cirrhosis significantly impacts the length of hospitalization. The effect of cirrhosis on the length of stay was the most prominent in the subgroup of viral CLD hospitalizations and accounted for an increase of 4.19 (95% CI [2.29, 6.33]) days, which the aforementioned reasons could explain. This relationship has not been examined frequently in the literature. However, several authors did report that patients with cirrhosis as a co-morbidity (irrespective of the main reason for hospitalization) had a significantly longer index hospital stay and total hospital charges compared with patients without cirrhosis [14,35]. On the other hand, in the presence of cirrhosis, patients with autoimmune liver diseases exhibited a lower average hospitalization length of −1.64 (95% CI [−4.57, 0.87]) days, which could be explained by higher mortality rates in end-stage liver disease. The wide posterior distribution of the estimated effect for “overlapping” liver disease reflects the heterogeneity in clinical states and outcomes of these patients. It is without doubt that the presence of cirrhosis in ArLD and viral hepatitis (and in general) involves more significant patient management and healthcare resource expenditure. Hence, the additional time spent in the hospital reflects additional disease burden.

## 5. Limitations

Since the actual rate of co-morbidities is unobserved and only the number of co-diagnoses is known, the physician is a possible confounder. Suppose the physician’s “effect” determines a significant portion of the length of stay. In that case, her vigilance in detecting and documenting co-morbidities and risk aversion will bias the estimate. The omission of such a variable is an essential source of unmodeled variation, which should be the target of future studies. In addition, given that our analysis is entirely based on the ICD-10 codes assigned by attending physicians, coding is susceptible to reporting bias due to various factors. However, when it comes to end-stage liver disease, it has been reported that ICD-10 codes for liver cirrhosis can be considered reliable and accurate [36]. Furthermore, because our dataset lacked unique patient identifiers for a substantial subset of the cohort (n = 1069), we utilized the individual hospitalization episode as our primary unit of analysis. Discarding these records to allow for patient-level clustering would have introduced severe selection bias; consequently, intra-patient correlation from repeated admissions remains unmodeled, which may result in slightly underestimated standard errors. Finally, we did not include hospitalizations due to MASLD, one of the most prevalent CLDs. One of the main reasons for that was the frequent and recent changes in nomenclature and classification, which are still not adequately represented throughout the ICD codes in our country and are, therefore, highly susceptible to miscoding. However, given that the current epidemiologic burden of MASLD is enormous and continuing to grow, with a predicted disease prevalence of 55.4% by 2040 [5], it is no surprise that MASLD is becoming the dominant liver disease worldwide. On the other hand, MASLD significance also reflects itself through numerous associated co-morbid conditions which significantly affect patients’ wellbeing. Therefore, the evaluation of MASLD’s rising prevalence, hospitalization trends and outcomes, together with estimating the effect of co-morbidities on both disease course and its outcome, would be of great significance and should be considered as the main research subject of future studies.

## 6. Conclusions

The expected hospital stay was the highest for the primary diagnosis of autoimmune liver disease, followed by ArLD. We identified the presence of cirrhosis and the number of co-diagnoses as factors that affect hospitalization length, together with hospitalization length regarding the CLD etiology. In addition, as the number of co-diagnoses accumulates, the length of hospital stay starts to increase dramatically and at a faster rate for infectious diseases than for hospitalizations due to CLD of other etiology. Our findings can inform medical practitioners and public health policy makers that there is an urgent need to detect liver disease at earlier stages and better manage associated co-morbidities.

## Figures and Tables

**Table 1:** Baseline demographic and clinical characteristics of the hospitalized study population, stratified by primary diagnosis. Continuous variables (hospitalization length, age, co-diagnosis burden) are presented as median (interquartile range), and categorical variables (gender, cirrhosis, discharge type) are presented as frequency (percentage). The total cohort (N = 3975) was driven largely by alcohol-related liver disease (ArLD) admissions, which featured a predominantly male demographic (90%), the highest median co-diagnosis burden (5), and the highest in-hospital mortality (16%, alongside overlap syndrome). Conversely, autoimmune liver disease was predominantly female (77%) and associated with the highest documented prevalence of cirrhosis (78%) but lower mortality (3.5%). Patients with viral liver disease exhibited the shortest median length of stay (5 days) and the lowest baseline co-diagnosis burden.

		Primary Diagnosis			
Characteristic	Overall, N = 3975	ArLD, N = 2164	Autoimmune, N = 601	Overlap, N = 62	Viral, N = 1148
Hospitalization (days) ^1^	9 (4, 17)	11 (6, 18)	12 (7, 21)	9 (5, 17)	5 (2, 10)
Age ^1^	58 (47, 65)	60 (53, 66)	56 (41, 65)	50 (42, 59)	50 (39, 62)
Gender, N (%)					
F	1113 (28%)	225 (10%)	462 (77%)	8 (13%)	418 (36%)
M	2862 (72%)	1939 (90%)	139 (23%)	54 (87%)	730 (64%)
Co-diagnosis ^1^	3 (0, 7)	5 (2, 9)	3 (1, 6)	7 (4, 9)	1 (0, 3)
Cirrhosis, N (%)	896 (23%)	258 (12%)	466 (78%)	14 (23%)	158 (14%)
Discharge type, N (%)					
Death	423 (11%)	353 (16%)	21 (3.5%)	10 (16%)	39 (3.4%)
Discharge	3552 (89%)	1811 (84%)	580 (97%)	52 (84%)	1109 (97%)

^1^—Median (IQR); ArLD, Alcohol-related liver disease.

**Table 2:** Expected average length of hospital stay stratified by primary liver disease diagnosis. Estimates and 95% credible intervals (CIs) are derived from the posterior predictive distributions of the primary Bayesian lognormal model, adjusting for age, co-diagnosis burden, and cirrhosis status. The model anticipates the longest average hospitalization for patients with a primary diagnosis of autoimmune liver disease (15.89 days), followed closely by alcohol-related liver disease (ArLD) (14.22 days). Conversely, hospitalizations primarily driven by viral hepatitis are expected to be notably shorter, averaging 8.83 days. Patients with overlap syndrome demonstrate a high expected length of stay (13.06 days) but exhibit the widest credible interval (10.52–17.0 days), reflecting greater clinical and observational heterogeneity within this subgroup.

	Expected Length of Stay		
Diagnosis	Estimate (Days)	CI 2.5%	CI 97.5%
Autoimmune	15.89	14.74	17.2
ArLD	14.22	13.68	14.79
Overlap	13.06	10.52	17
Viral	8.83	8.27	9.43

CI, credible interval.

## Data Availability

The datasets used and analyzed during the current study are available from the corresponding author on reasonable request due to ethical reasons.

## Supplementary Materials

The following supporting information can be downloaded at: https://www.mdpi.com/article/10.3390/clinpract16030057/s1, Figure S1. Markov Chain Monte Carlo (MCMC) trace plots for selected model parameters. This figure displays trace plots for select parameters, showing the sampling progression across iterations (x-axis) and the sampled values (y-axis). Different colors represent separate chains, illustrating their convergence and the effective exploration of the parameter space, which is essential for confirming the stability and reliability of the analysis. Figure S2. Empirical cumulative distribution function (ECDF) of the empirical probability integral transform (PIT) values. The x-axis represents the PIT values, ranging from 0 to 1, while the y-axis shows the cumulative proportion of observations. A closely aligned ECDF curve to the diagonal line indicates a well-calibrated model, suggesting that predictions come from the same distribution as the observed outcomes.

## Abbreviations

ArLD	Alcohol-related liver disease
CI	Credible interval
CLD	Chronic liver disease
COVID-19	Coronavirus disease
DAG	Directed acyclic graph
ESS	Effective sample size
ICD-10	10th revision of the international classification of diseases
LOO-PIT	Leave-one-out probability integral transform
MASLD	Metabolic dysfunction-associated steatotic liver disease
MCMC	Hamiltonian Markov Chain Monte Carlo
PSIS	Pareto-smoothed importance sampling leave-one-out cross-validation
WHO	World Health Organization
