# Supplementary material for: Hospitalization Trends Due to Chronic Liver Diseases: Vicious Circle of Co-Morbidities and Hospitalization Length
Source: Clin Pract. 2026 Mar 6;16(3):57. doi: 10.3390/clinpract16030057 (PMC13025581; doi:10.3390/clinpract16030057)
Supplement: Supplementary file 1 [file clinpract-16-00057-s001.zip › clinpract-4151716-supplementary.pdf]

Supplementary Materials: Model Diagnostics

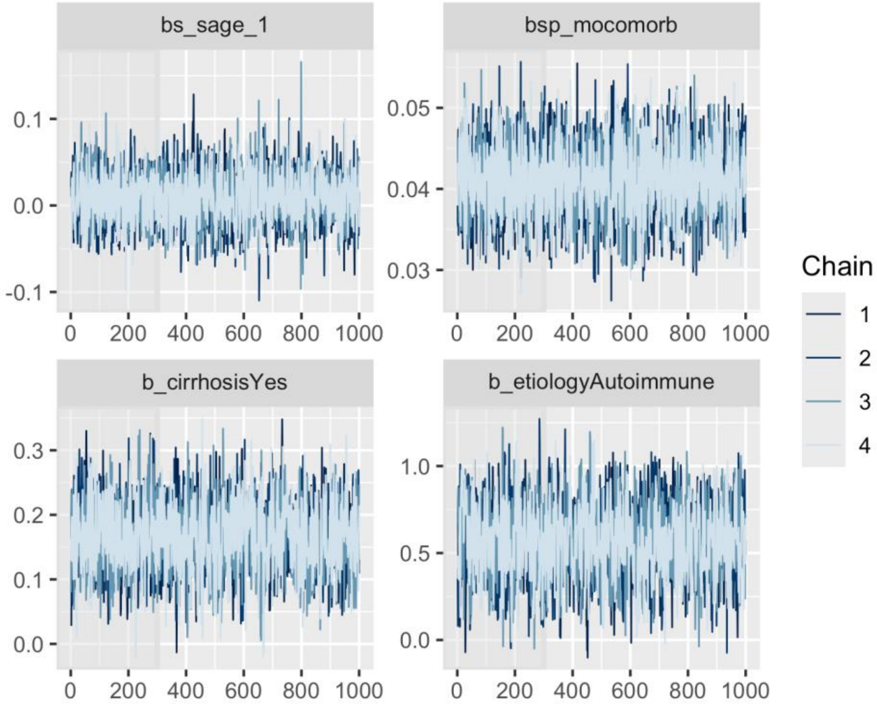

Figure S1. Markov Chain Monte Carlo (MCMC) trace plots for selected model parameters. This figure dis-plays trace plots for select parameters, showing the sampling progression across iterations (x-axis) and the sampled values (y-axis). Different colors represent separate chains, illustrating their con-vergence and the effective exploration of the parameter space, which is essential for confirming the stability and reliability of the analysis.

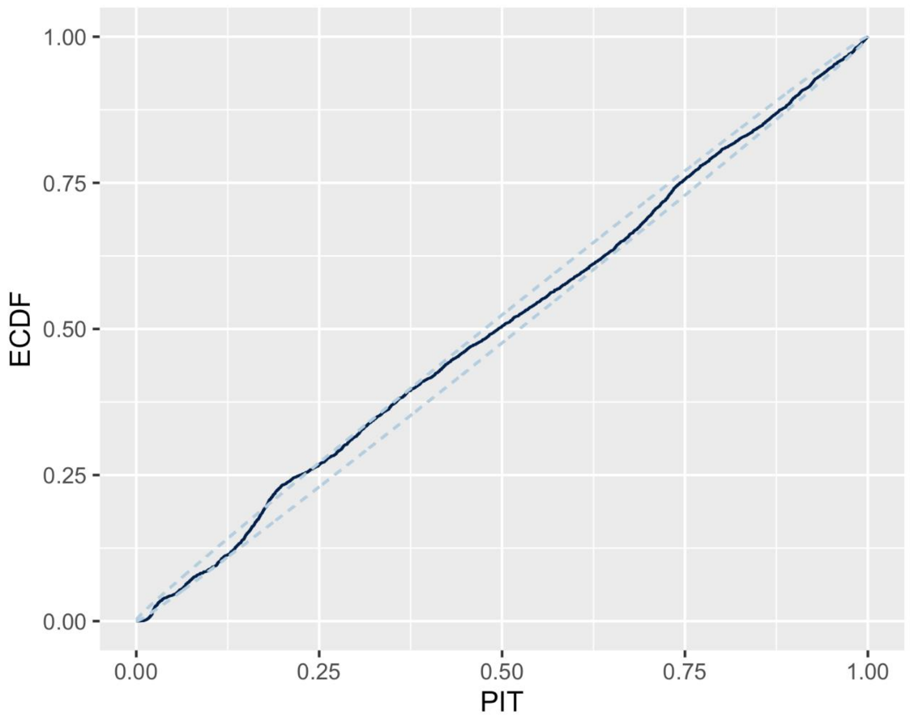

Figure S2. Empirical cumulative distribution function (ECDF) of the empirical probability integral transform (PIT) values. The x-axis represents the PIT values, ranging from 0 to 1, while the y-axis shows the cumulative proportion of observations. A closely aligned ECDF curve to the diagonal line indicates a well-calibrated model, suggesting that predictions come from the same distribution as the ob-served outcomes.
